# A curve-crossing model to rationalize and optimize diarylethene dyads†
†Electronic supplementary information (ESI) available: Analytic expression for the energies of Fig. 3, complete table of tilt values for the hetero-dyads, orbital considerations for selected systems. See DOI: 10.1039/c5sc01960e


**DOI:** 10.1039/c5sc01960e

**Published:** 2015-06-29

**Authors:** Benjamin Lasorne, Arnaud Fihey, David Mendive-Tapia, Denis Jacquemin

**Affiliations:** a Institut Charles Gerhardt Montpellier , UMR 5253 , CNRS-UM , CTMM , Université Montpellier , CC 1501, Place Eugène Bataillon , 34095 Montpellier , France . Email: david.mendive-tapia@univ-montp2.fr; b Chimie Et Interdisciplinarité, Synthèse, Analyse, Modélisation (CEISAM) , UMR CNRS no. 6230 , BP 92208 , Université de Nantes , 2, Rue de la Houssinière , 44322 Nantes Cedex 3 , France . Email: denis.jacquemin@univ-nantes.fr; c Institut Universitaire de France , 103 bd St. Michel , 75005 Paris Cedex 5 , France

## Abstract

Going from photochromic compounds presenting a single switchable function to multi-addressable photochromic multimers remains an extremely difficult task notably because the interactions of several photochromic units through a linker generally result in a substantial loss of photoactivity. Due to their size and the intrinsic complexity of their electronic structure, coupled photochromes also constitute a fundamental challenge for theoretical chemistry. We present here an effective curve-crossing model that, used in connection with easily accessible *ab initio* data, allows a first understanding of the difficulty to obtain efficient multiphotochromes. Indeed, we demonstrate that extra crossing points, specific to multiphotochromes, have to be passed to ensure reactivity. In addition, the proposed approach allows the definition of an intuitive tilt criterion that can be used to screen a large number of substitution patterns and hence help in the design of new compounds, an aspect that is also developed here. The compatibility of this tilt criterion with previously proposed static Franck–Condon parameters is discussed as well.

## Abstract 2

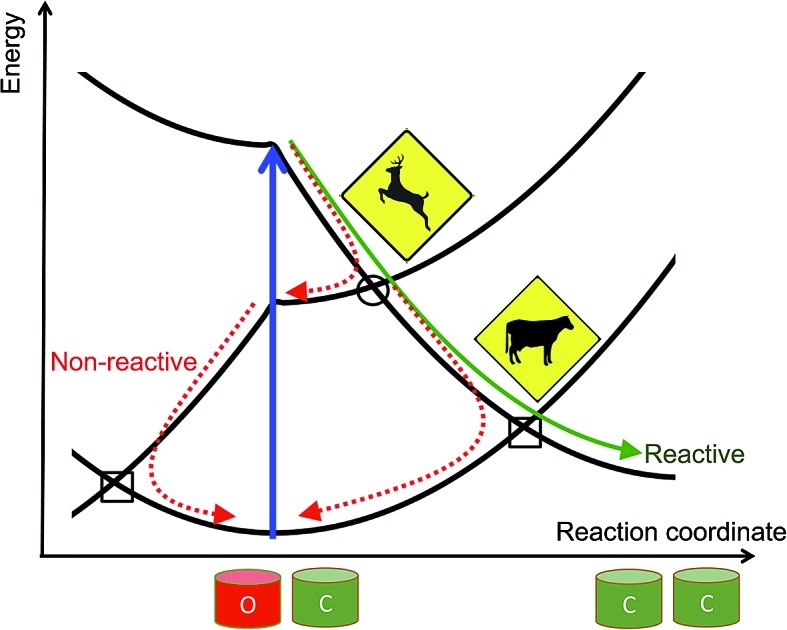
Extra crossing points play a key role in the photochemistry of diarylethene dyads.

## 1 Introduction

**Fig. 1:**
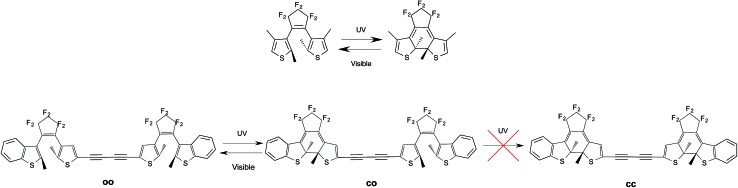
Top: representation of a typical DAE with the o (left) and c (right) isomers. Bottom: experimental evolution for the DAE dyad studied in ref. 6: the second cyclization is not observed.

Photochromes are molecules able to switch from one isomeric form to another after absorption of light. Amongst all classes of photochromic compounds synthesised to date, diarylethenes (DAEs) occupy a privileged spot as they stand as the most effective (and most investigated) thermally-stable photochromes.^1^–^4^ As illustrated in Fig. 1, under irradiation with UV light, DAEs can go from a colorless and poorly conjugated open (o) isomer to a colored and extensively conjugated closed (c) isomer. The basic photochemical process taking place during the transformation of o-DAE into c-DAE, is a Woodward–Hoffman type cyclization of the central *cis* hexatriene unit into a cyclohexadiene structure. In the most-stable open form, the two thiophene rings are anti-parallel to each other and perpendicular to the central perfluoro bridge, whereas all π-bonds are nearly coplanar in the closed isomer. This substantial difference in electronic structure results a very large optical contrast: c-DAEs typically absorb light at *ca.* 500–600 nm, whereas the first absorption bands of o-DAEs are located at *ca.* 300 nm. As many DAEs are also fatigue-resistant,^5^ they can in principle be used to store a bit of information, the o (c) isomer acting as a “0” (“1”) memory state.

To go further, molecules encompassing several switchable units have been proposed, notably DAE dyads.^7^ The experimental outcomes were however rather frustrating. On the one hand, in systems in which the DAE subunits are almost non-interacting, *e.g.*, when a non-conjugated linker is used to bind the two DAEs, photochromism tends to be conserved but the visible absorption bands of the mixed closed–open (co) and fully-closed (cc) isomers peak at nearly the same wavelength, making it difficult to distinguish the two isomers with cheap spectroscopic techniques.^8^–^12^ On the other hand, strongly-coupled DAE dyads tend to present partial photochromism only: one can go from the oo to the co isomer but prolonged irradiation does not yield the expected cc structure.^13^ This is illustrated at the bottom of Fig. 1 for one specific dyad, but this holds for many other derivatives.^6^,^13^–^17^ In these situations, the DAE multimer behaves like a new partially reactive monomer and is thus a rather unexciting system.

To explain this loss of photochromism, energy transfer (ET) between the different subunits was invoked.^13^,^18^,^19^ The idea is that the irradiation of the open form in the hybrid co isomer yields a co* state that is rapidly deactivated by ET leading to c*o. While this interpretation is convenient and chemically intuitive, there is, to the best of our knowledge, no theoretical background supporting that interpretation. It is indeed possible to apply refined multi-reference wavefunction theories to explore the photochromism of isolated DAEs,^20^–^26^ but they are in practice not applicable to DAE dyads. For this reason, the present theoretical state-of-the-art is to use Time-Dependent Density Functional Theory (TD-DFT) to determine the nature of the excited-state in the Franck–Condon (FC) region for all isomers.^27^ Such a crude and static procedure was able to explain several experimental outcomes,^28^–^30^ but as it is completely blind in the actual photochemical region, it incorrectly predicts several experimental events.^28^,^31^ For instance, Staykov and collaborators explored the properties of two DAE separated by a sexithiophene linker,^32^ and by considering several charged states, they indicated an orbital control of the photochromism. We have recently used a similar approach to investigate a large series of dimers,^33^ and could define optimal substitution patterns providing an (FC) excited-state with the ideal topology, but no proper assessment on how the photoreactivity takes place after the vertical transition could be performed. To try to bypass this limitation, we provide here the first model allowing a direct rationalisation of the photoreactivity of DAE dyads. This model is based on electronic-state correlation diagrams built from the energy profiles of the isolated DAE units and considers the weak-coupling regime (spectator bridge). We focus on the occurrence of curve crossings along ring-closing/opening pathways, which could decrease the yield of some of the products by inducing branching in the transfer of population. Despite its limits (weak-coupling), this model allows a fundamental understanding of the difficulty of designing efficient DAE dyads and provides hints at the most adequate substitution patterns. In that sense, it is complementary to the previously proposed “orbital TD-DFT” approach.

## 2 Methods

In this Section, we present our model. By convention, we note A and B the two DAEs of the A–X–B dyad, where X is the linker. As stated above, c and o indicate the isomeric state of the DAE.

### 2.1 Isolated DAE

**Fig. 2:**
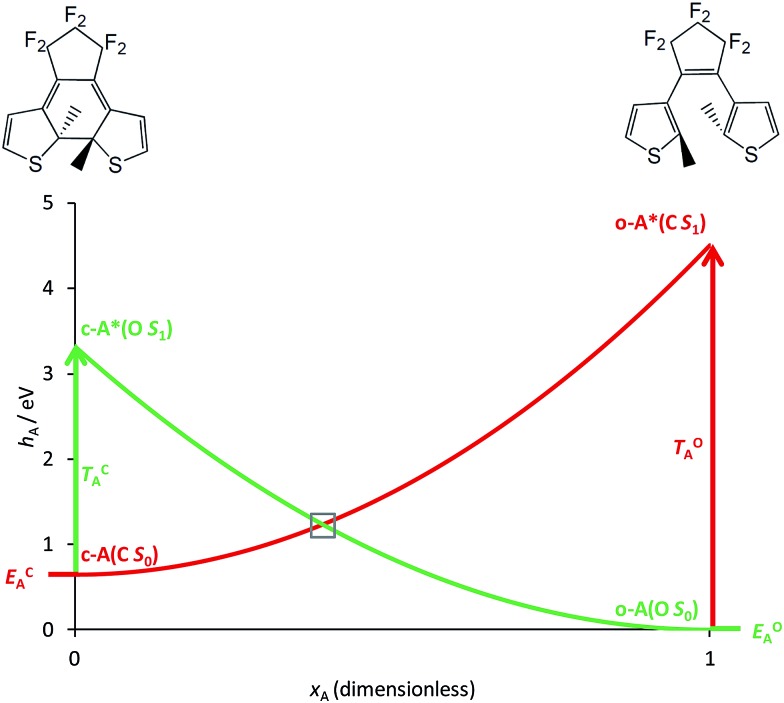
Simplified correlation diagram for a single DAE unit A (red: state C; green: state O). *x*_A_ is the dimensionless reaction coordinate linking the open (*x*_A_ = 1, rhs on the Figure) and closed (*x*_A_ = 0, lhs on the Figure) isomers, *h*_A_ defines the relative energies (in eV). The energy parameters, corresponding to an isolated DAE displayed on top of the Figure, have been taken in ref. 33.

The basic photoactive unit of DAEs is the cyclohexadiene/hexatriene system, a textbook prototype for electrocyclisations/cycloreversions. Theoretical studies have shown that the *S*_0_ and *S*_1_ potential energy surfaces of DAE cross along a seam of *S*_1_/*S*_0_ conical intersection (CI), which explains the efficient photoswitching mechanism that takes place through an ultrafast internal conversion.^21^–^23^,^25^ The *S*_2_ state also plays a role, as it interacts with the *S*_1_ state through an ionic/covalent mixing that changes the electronic character of *S*_1_ when going from the FC region to the *S*_1_/*S*_0_ CI.^21^,^25^ Hereafter, we will ignore this latter aspect and simply consider that each DAE unit is characterised by two electronic states, *S*_0_ and *S*_1_, the ground state and the first bright excited state, respectively. A simplified correlation diagram for these adiabatic states along a ring-opening/closing reaction coordinate can thus be built, where a valence-bond-type (VBT) state,^34^ denoted C, correlates the *S*_0_ closed isomer c-A to the *S*_1_ open isomer o-A* while a second VBT state, denoted O, correlates the *S*_0_ open isomer o-A to the *S*_1_ closed isomer c-A* (see Fig. 2). A similar notation will be used for the second DAE unit B in the following. We note that this correlation between the excited-state of the closed form and the ground-state of the open isomer (and *vice-versa*), was also found when examining the topology of the frontier molecular orbitals obtained through DFT calculations.^35^,^36^

### 2.2 DAE dyads

As explained in the Introduction, the closed–closed form, that is (c-A)–X–(c-B), is elusive in several cases. In this work, we aim to propose a general explanation of this outcome. To this end, we build a model based on a comparison of the intrinsic properties of the DAE units, irrespectively of the chemical nature of the bridge in the small-coupling limit. Our description is based on a separable zero-order electronic Hamiltonian (*H*^0^) defined as the sum of the clamped-nucleus electronic Hamiltonians of the two isolated DAE units (*h*_A_ and *h*_B_),

1 *H*^0^ (*x*_A_, *x*_B_) = *h*_A_ (*x*_A_) + *h*_B_ (*x*_B_)

Of course, in practice the isolated DAE units are in fact the hydrogen-capped moieties, A–H and H–B, so to obtain closed-shell species rather than radical fragments. As we focus our description on the electronic π-system, we can ignore this subtlety for the time being. Each reaction coordinate, *x*_A_ or *x*_B_, will be defined as a dimensionless and scaled parameter so that it is respectively 0 for the closed form and 1 at the open form (see Fig. 2). In addition, we assume that the reaction coordinates are curved in such a way that the reaction path goes through the *S*_1_/*S*_0_ CI along a direction that lifts degeneracy. In other words, we assume that *x*_A_ or *x*_B_ present large enough components along the branching-plane vectors in the vicinity of the CI. Using the states C and O defined above as a basis set, the diagonal entries of the one-unit Hamiltonian can be expressed as quadratic functions of the reaction coordinate,2
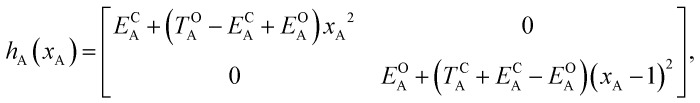



The *E*-parameters correspond to the absolute energies, whereas the *T*-parameters are the vertical transition energies (see Fig. 2). By construction, at both points *x*_A_ = 0 (closed) and *x*_A_ = 1 (open), they respectively satisfy3
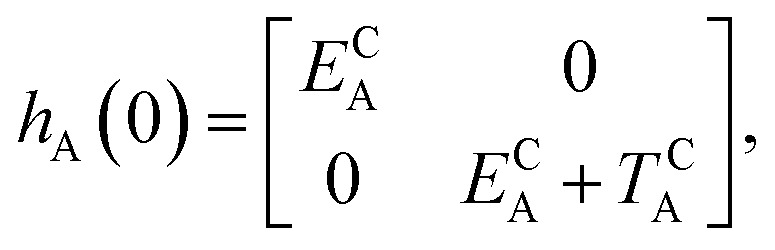

4
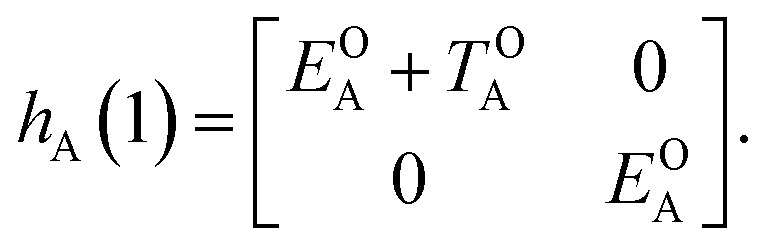



For simplicity, we set *E*OA = 0, that is, we use the ground-state energy of the most stable open isomer as reference. We also introduce *D*COA = *E*CA – *E*OA, the difference between the ground state energies of the two isomers. This term is positive for all systems investigated here. The three preceding equations now read,5
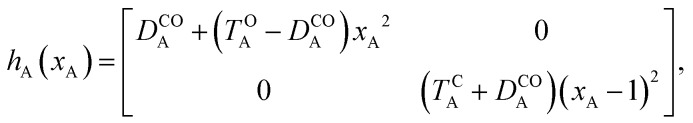

6
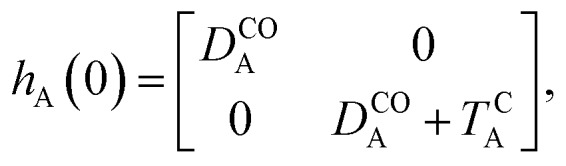
and7
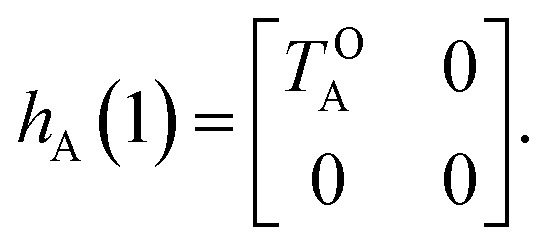
Similar expressions hold for B.

Now, from this simple additive description, we can build four singlet “direct-product states” based on the states C and O of the fragments: CC, CO, OC, and OO, where the first (second) label indicates the VBT state of the A (B) DAE unit. When such states occur to be excited states, according to the geometry of the dyad, they are characterised by excitations localized on either or both of the two fragments. Here, we assume that these four states are dominated by electronic transitions between photochromic orbitals that is the orbitals involved in the photoreaction (see ref. 27 for a definition of such orbitals). However, they can be embedded in a set of other low-lying excited states ignored in this work. In other words, CC, CO, OC, and OO are not necessarily zero-order approximations of *S*_0_, *S*_1_, *S*_2_, and *S*_3_ but rather of the states that are expected to play the most significant role in the photochromic activity of DAE dyads.^37^

The zero-order electronic Hamiltonian matrix of the dyad, *H*^0^ (*x*_A_, *x*_B_) is diagonal, as we neglect direct coupling, and its diagonal elements read:

8 *H*0CC(*x*_A_, *x*_B_) = *D*COA + *D*COB + (*T*OA – *D*COA)*x*_A_^2^ + (*T*OB – *D*COB)*x*_B_^2^,

9 *H*0CO(*x*_A_, *x*_B_) = *D*COA + (*T*OA – *D*COA)*x*_A_^2^ + (*T*CB + *D*COB)(*x*_B_ – 1)^2^,

10 *H*0OC(*x*_A_, *x*_B_) = *D*COB + (*T*CA + *D*COA)(*x*_A_ – 1)^2^ + (*T*OB – *D*COB)*x*_B_^2^,

11 *H*0OO(*x*_A_, *x*_B_) = (*T*CA + *D*COA)(*x*_A_ – 1)^2^ + (*T*CB + *D*COB)(*x*_B_ – 1)^2^.

where we have set *E*OA + *E*OB = 0, as reference energy.

## 3 Computational details

To provide input to the above-described model, we relied on calculations of the properties of the (hydrogen-capped and isolated) DAE performed with DFT and TD-DFT, that respectively give access to the ground-state energies of the open and closed forms (*E*^O^ and *E*^C^) and to the corresponding vertical transition energies (*T*^O^ and *T*^C^). First, the ground-state geometry of each system was fully optimized with the help of a global hybrid functional, namely PBE0,^38^,^39^ and vibrational frequencies were computed at the same level of theory to ensure that geometries correspond to global minima. Then the transition energies to the low-lying excited-states were determined at the TD-DFT level with the CAM-B3LYP^40^ range-separated hybrid functional. Similar combinations of methods have been widely used for DAE monomers and multimers.^28^,^30^,^41^ For all steps the 6-31G(d) atomic basis set was used, as it is sufficient to determine the relative energies we are looking for. All DFT/TD-DFT calculations were conducted in the gas phase, with the Gaussian 09 package.^42^

## 4 Results and discussion

### 4.1 Understanding the complexity of multiswitches

**Fig. 3:**
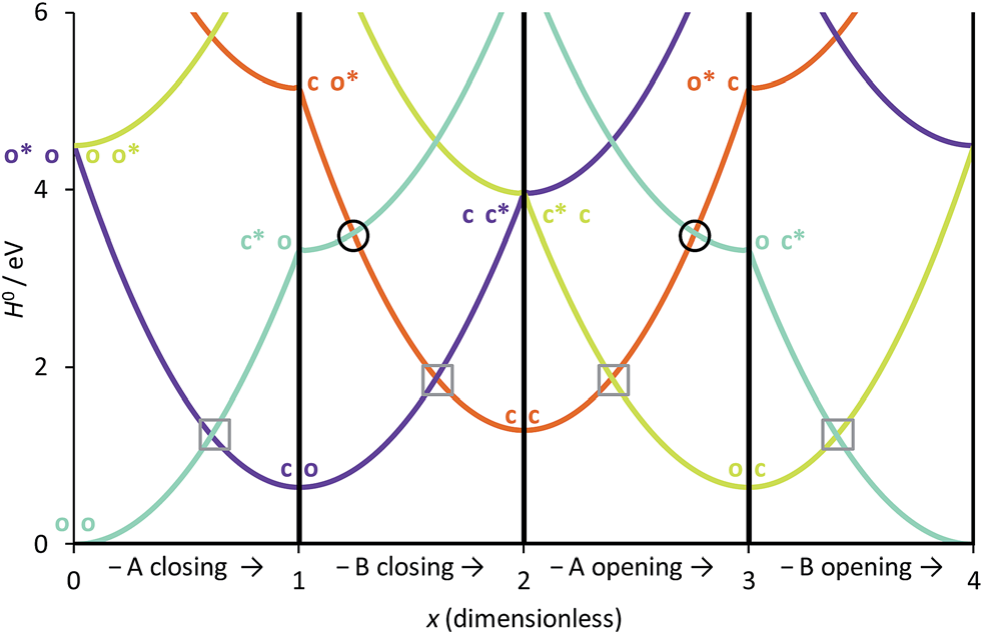
Simplified cyclic reaction path from (o-A)–X–(o-B) to (c-A)–X–(o-B) to (c-A)–X–(c-B) to (o-A)–X–(c-B) and back to (o-A)–X–(o-B). Blue curve: OO state; purple curve: CO state; orange curve: CC state; green curve: OC state. The energy parameters correspond to a symmetric dyad made of the two DAEs displayed in Fig. 2. The *x*-coordinate corresponds to a cyclic “square path” relating the four possible minimal structures corresponding to all open/closed combinations.^43^*x* was chosen so that one goes from the fully open structure at *x* = 0 to the fully closed structure at *x* = 2 (and hence its sign is different from that of *x*_A_ of Fig. 2).^43^ Note that *x* = 0 and *x* = 4 correspond to the same point, namely the fully open dyad, whereas *x* = 1 and *x* = 3 correspond to mixed closed/open structures.

We have used eqn (8)–(11) and the same energy parameter as the one used for the isolated DAE of Fig. 2 to obtain a first grasp on the working mechanism of DAE dyads. The results are plotted in Fig. 3. The corresponding expressions of the diagonal elements are given in the ESI (Table S-1).† On Fig. 2, the grey square indicates the crossing between the VBT states C and O along the reaction coordinate. In the weak coupling limit, such crossings between the ground state and the first excited state also occur in the dyad and are not different in nature from those involved in each single DAE unit. For this reason they are indicated in a similar way in Fig. 3. We will denote this type of crossings as *normal* crossings in the following. In contrast, the black circles in Fig. 3 indicate *extra* crossings between the first and the second excited states, that were absent in the single DAE case and need to be crossed to go from the co to the cc isomer. This extra complexity is most probably a key to explain why several DAE dyads cannot form the closed–closed isomer. Interestingly, we note that the *extra* crossings open a path allowing to go from c*–X–o to c–X–o*, which is consistent with the qualitative *ET* explanation given previously in experimental works.^13^,^18^,^19^ In short, the photochemical pathways connecting o–X–o to o–X–c or c–X–o are simple and remain very similar to these of the isolated DAE units, indicating that the first electrocyclization of the dyads should be similar to the one encountered in isolated DAEs. However, the photochemical pathways connecting o–X–c or c–X–o to c–X–c are more complicated and potentially less efficient because they involve both *normal* and *extra* crossings. This is well in line of the experimental trends: one can easily close the first DAE units in multimers, but not the others.^7^

### 4.2 Enhancing the efficiency of the second photoreaction

**Fig. 4:**
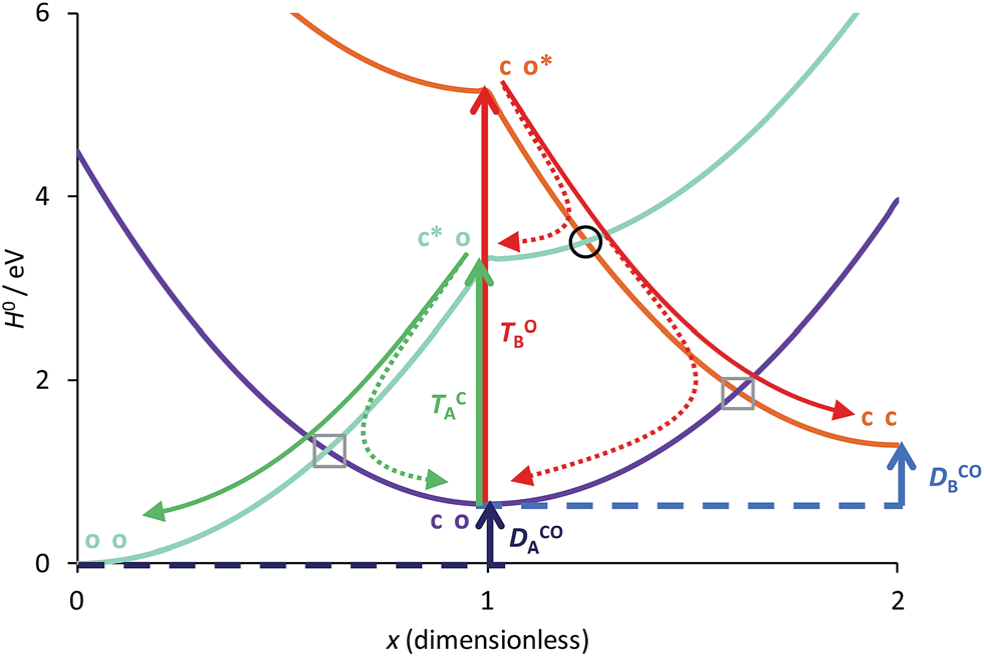
Comparison between the formation of (o-A)–X–(o-B) from (c-A)*–X–(o-B) through a single crossing (grey square) and the formation of (c-A)–X–(c-B) from (c-A)–X–(o-B)* through a sequence of two crossings (black circle and grey square). The energy parameters are the same as in Fig. 3. The full (dashed) arrows indicate reactive (unreactive) photochemical pathways. On this Figure, the doubly open dyad is at the left hand-side (*x* = 0).

To continue our analysis and in the perspective of molecular design, we provide in Fig. 4 a focussed view of the main phenomena in the 0 ≤ *x* ≤ 2 region, so that we consider that DAE A (B) will be the first (second) to close. Together Fig. 3 and 4 allow one to examine how changing the values of the total and transition energies with chemical substitution does affect the characteristics of the *extra* crossings. To avoid making chemically unrealistic predictions, we remind that, for DAE, the vertical transition energy of the open form is in most cases larger than that of the closed form (*T*OA/B > *T*CA/B) whereas the closed form is less stable in the ground state than the open form (*E*CA/B > *E*OA/B, meaning that *D*COA/B > 0). From Fig. 4, it seems obvious that increasing *T*OB should go together with an improvement of the yield of formation of the closed–closed dyad because the system will have extra kinetic energy when arriving at the *extra* crossing (black circle) and the local slope at the crossing should favour an efficient radiationless transition (ballistic behaviour favouring a diabatic crossing). In other words, larger *T*OB should decrease the efficiency of the ET path leading from co* to c*o (see Fig. 4). Decreasing the amplitude of *D*COB should have a similar effect. Note that such effects are expected to hold also for the *normal* crossings (grey squares) that are probably not limiting factors for dyads.

Other energy parameters may also influence the outcome and more systematic indicators are required. To this end, let us consider the extra crossing between CC and OO, the two states correlating the oo and cc isomers in the ground-state, over the interval displayed in Fig. 4. The possible values of *x*_B_ for which *H*0OO(0, *x*_B_) = *H*0CC(0, *x*_B_) are given by,12
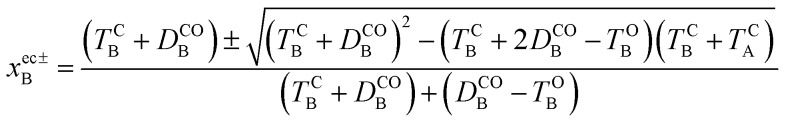
where ec stands for extra crossing. The value lying in the interval of interest (between 0 and 1 for *x*ec±B, meaning between 1 and 2 for *x* in Fig. 4)^43^ is *x*ec–B and it is used in the following. In addition, the topography of the extra crossing is peaked and significant population branching is expected. The tilt of the crossing is thus a good indicator of the efficiency of the transfer of population. The average gradient reads:13
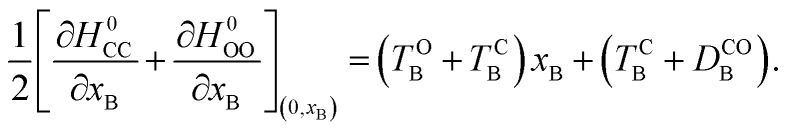



A positive value at the crossing point indicates an average force pointing toward *x*_B_ = 0. Increasing its magnitude should thus favour formation of the closed–closed dyad (c-A)–X–(c-B) by enhancing the ballistic behaviour of the system as it passes through the *extra* crossing.

### 4.3 Screening DAE dyads

**Fig. 5:**
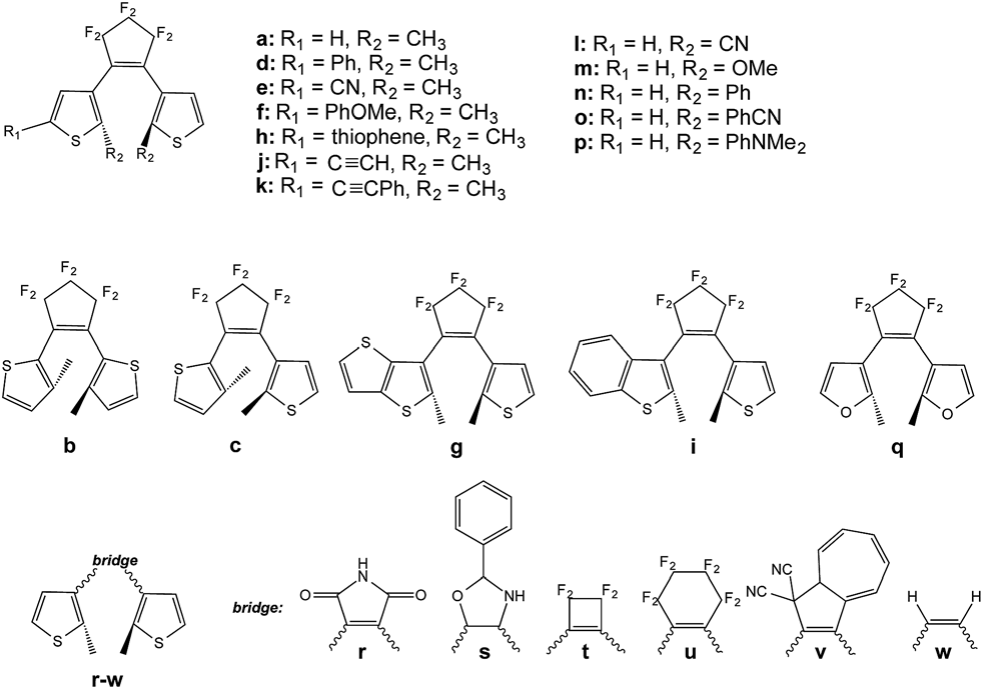
Systems investigated in the screening process.

We now treat the 23 DAEs presenting various chemical substitutions and displayed in Fig. 5. This set contains DAEs with various substituents on both the α position of the thiophene rings and the reactive carbon atoms, inverse and normal DAE, as well as a series of bridges that were used previously.^44^

Let us start by considering homo-dyads (B–X–B). The results are listed in Table 1. For all studied systems, *x*ec–B fall in the quite narrow 0.7–0.9 range, *e.g.*, it is 0.76 for the seminal DAE dimer (**a**–X–**a**). By contrast, the tilt criterion is much more sensitive to the chemical nature of the DAE, it ranges from 0.86 to 2.66 eV (it is 2.13 eV for the reference **a**–X–**a** dyad). A general trend emerges: adding substituents on the reactive carbon atoms (**l–p** series) yields a smaller tilt criterion, which should induce a less efficient reaction to the *extra* crossing. This contrasts with the study of the topology of the orbitals at the FC point that indicated that this substitution of the reactive carbon atoms can be very useful (but in a strong coupling case),^33^ illustrating the difficulty to simultaneously optimize all parameters. We underline that the tilt criterion focuses on the reactivity at the crossing region, whereas orbital topology criteria focus on the nature of the FC point, and these two criteria are therefore complementary (see next Section). Keeping constant the perfluorocyclopentene bridge, the replacement of the thiophene rings by a thieno-thiophene group (**g**) or by a furan cycles (**q**) appear as the most effective options to increase the tilt, though the effect is rather moderate compared to the standard a–X–a dimer (+7% and +15%, respectively). To the very best of our knowledge, the use of furan rings in DAE multimers was never assessed experimentally, but furan-based DAE monomers present large cyclization quantum yields, similar to the one obtained with thiophene-DAEs.^45^–^47^ The largest tilt are reached for **w** and **u**. However, the former is not a chemically interesting option. Monomers of DAE using the latter cyclohexene bridge have been synthesized, but were found to be less effective that the corresponding perfluororcyclopentene structures.^48^,^49^ On balance, the maleimide group (**r**) is probably the most pertinent “bridge” option to increase the tilt. In DAE monomer, this maleimide bridge yields however less efficient cyclization than the well-known perfluorocyclopentene bridge, especially in polar environments.^50^–^52^

**Table 1:** Energy parameters (in eV), position of the *extra* crossing (dimensionless) and tilt criterion (in eV) for various symmetric DAE dyads B–X–B. See Fig. 5 for the corresponding structures

Compound	*D* CO B	*T* C B	*T* O B	*x* ec– B	Tilt
**a**	0.65	2.68	4.51	0.76	2.13
**b**	0.97	3.19	4.08	0.86	2.09
**c**	0.79	2.93	4.30	0.81	2.10
**d**	0.59	2.52	4.33	0.75	2.05
**e**	0.72	2.48	4.53	0.72	1.87
**f**	0.56	2.50	4.23	0.76	2.06
**g**	0.45	2.61	4.25	0.78	2.28
**h**	0.55	2.46	4.22	0.75	2.03
**i**	0.53	2.66	4.32	0.78	2.24
**j**	0.65	2.50	4.42	0.74	1.97
**k**	0.62	2.40	4.11	0.75	1.87
**l**	1.19	2.78	4.17	0.77	1.42
**m**	0.70	2.58	4.24	0.76	1.92
**n**	1.36	2.56	4.21	0.72	0.99
**o**	1.35	2.59	4.12	0.74	1.01
**p**	1.35	2.47	3.88	0.74	0.86
**q**	0.58	2.98	4.49	0.81	2.46
**r**	0.35	2.73	3.48	0.88	2.38
**s**	0.56	2.97	4.25	0.83	2.43
**t**	1.11	2.62	4.61	0.72	1.46
**u**	0.35	2.83	4.37	0.80	2.61
**v**	0.58	2.59	3.53	0.84	1.98
**w**	0.44	2.93	4.79	0.78	2.66

Given these results, we have also evaluated the **qr** case (maleimide bridge, furan rings). Such a **qr**–X–**qr** dyad presents a tilt of 2.68 eV, the largest of the series (+26% improvement compared to a–X–a). As we show in the following section, the topologies of the associated molecular orbitals also indicate a possible second cyclization, and this system might be worth an experimental try. Nevertheless, we note that in the specific case of a symmetric **qr** dyad presenting an ethynyl linker (see next Section), the *S*_1_ and *S*_2_ states of the closed–open isomer are energetically close (2.62 eV and 3.33 eV, a difference of 0.71 eV to be compared to *ca.* 2.0 eV in most dyads) indicating that the extra crossing takes place close to FC point, which may be detrimental for the cyclization process.

The general trends noted above correlate to a large extent with the value of *D*COB. Increasing the stability difference seems unfavourable to forming the closed–closed isomer. The *extra* crossing is less tilted, which implies more branching between the two channels. This also affects the *normal* crossing in a similar way and both effects tend to play in the same direction. A good solution to favour formation of cc seems to stabilise the closed form.

After this discussion of symmetric dyads in which *T*CB = *T*CA, we analyse the impact of using two different DAEs. Of course, the synthesis of asymmetric dyads is more challenging, but examples exist in the literature.^15^,^53^ We have investigated the tilt for all A/B combinations and the results are given in Table S-2† in the ESI.† In that Table, we consider that the A DAE is the first to react, so that the closed–open isomer contains a closed A and an open B DAE. Increasing *T*CA – *T*CB favours the formation of the cc structure. Interestingly, if B is the standard DAE structure, **a**, the best candidate is to select an inverse DAE (**b**) as second photochrome, with a tilt criterion of 2.62 eV. From the data of Table S-2,† it is clear that significantly exceeding this value of tilt is difficult [the largest figure, 2.95 eV, is obtained with (c-**b**)–X–(o-**u**)], but many substitution patterns yield small tilts, close or even below 1 eV. As for the homo-dyads of Table 1, this is the case for hetero-dyads of DAEs substituted at the reactive carbon atoms. Often, but not always, improving the yield of formation of (c-A)–X–(c-B) from (c-A)–X–(o-B)* often hinders that from (o-A)*–X–(c-B), as both pathways are no longer equivalent. For instance, the 2.62 eV value noted above goes down to 1.52 eV is one considers a dyad with an open **b** and a closed **a**, rather than the reverse. Clearly, it is therefore of prime importance to determine which DAE cyclizes the first in such asymmetric dimers.

### 4.4 Considering both tilt and orbital topology criteria

**Fig. 6:**
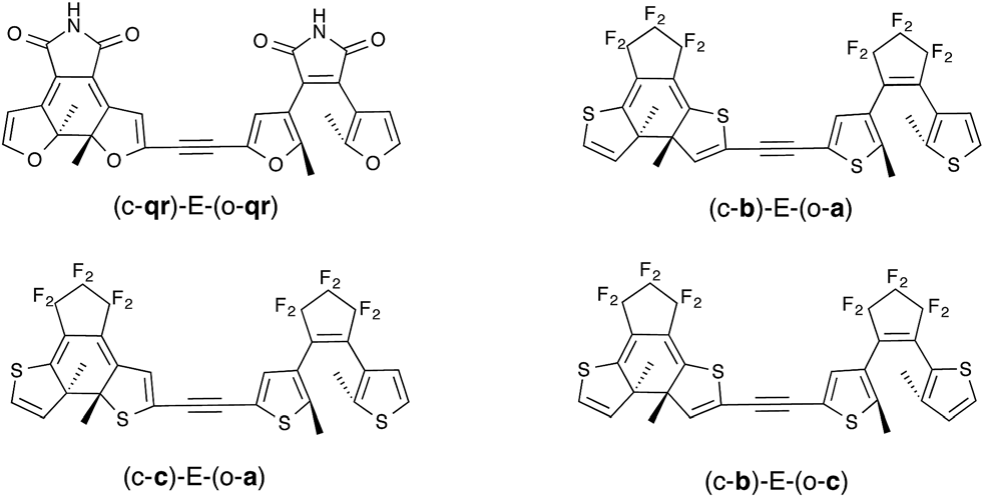
Ethynyl-linked dimers considered. Only the relevant closed-open form is shown.

As stated in the Introduction, the study of the topology of the virtual orbitals involved in the key electronic transitions was also used previously to assess the photo-activity of the closed–open isomer of multi-DAEs. This procedure, detailed elsewhere,^27^,^33^ also provides indications regarding the DAE unit that closes the first in the open–open form of hetero-dyads. This latter aspect is quite reliable, as one “only” needs to reproduce the energetic ordering of the first two excited-states that are localized on the A and B DAE in an asymmetric dimer, a task for which TD-DFT is well suited. This type of orbital analysis, limited to the FC point, is totally blind in the CI region but allows to model strongly-coupled multimers. In this Section, we use some of the best combinations obtained with the tilt criterion and assess their properties in dyads coupled through an ethynyl (E) linker (see Fig. 6). This π-conjugated linker was selected because it is known to be particularly problematic.^13^

As seen in Fig. 6, we have considered a symmetric dimer built with two **qr** photochromes, as well as all asymmetric combinations of normal (**a**), inverse (**b**) and hybrid (**c**) DAEs. For these asymmetric dyads, we have first determined the unit undergoing the first cyclization in the fully-open isomer. Therefore the closed–open structures shown in Fig. 6 are the results of this first analysis (see the ESI† for optical properties and relevant orbitals of the open–open forms). The tilt criterion for these structures are large: 2.68 eV for (c-**qr**)–E–(o-**qr**), 2.62 eV for (c-**b**)–E–(o-**a**), 2.37 eV for (c-**c**)–E–(o-**a**) and 2.36 eV (c-**b**)–E–(o-**c**).

**Fig. 7:**
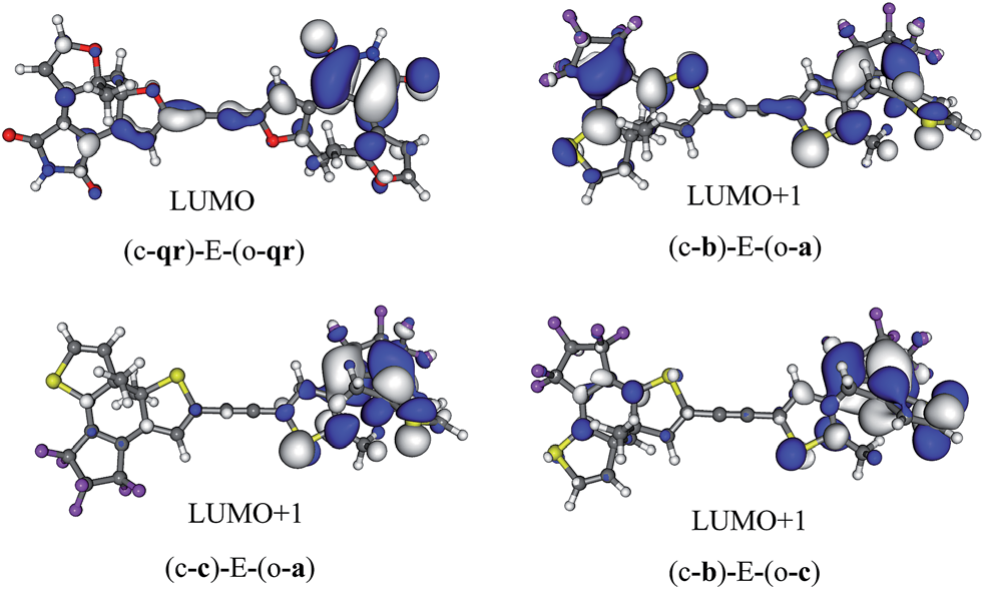
Photochromic virtual orbitals for the asymmetric dimers.

The symmetric (c-**qr**)–E–(o-**qr**) dyad presents a LUMO showing a clear bonding interaction between the reactive carbon atoms (see Fig. 7). This orbital is strongly involved (72% overall, see the ESI†) in the rather intense *S*_0_ → *S*_2_ transition peaking at 373 nm and relevant for ring-closure. Following the orbital criterion, this dyad is therefore expected to yield the fully closed form. Using the **qr** monomers, we have also tested other conjugated linkers and similar results were obtained (see the ESI†).

All three asymmetric dyads present a LUMO+1 as the first virtual orbital with a photochromic topology (see Fig. 7), a typical outcome.^27^ TD-DFT reveals that this orbital is not accessed by the same kind of transitions for the three compounds. In (c-**b**)–E–(o-**a**), it is populated (17% of the total) by the very intense *S*_0_ → *S*_2_ transition (*f* = 0.81) at 310 nm. In both (c–**c**)–E–(o-**a**) and (c-**b**)–E–(o-**c**) the LUMO+1 is only involved in transitions below 300 nm, but the efficiency of the transition is larger for the latter system (see the ESI†). In short, (c-**qr**)–E–(o-**qr**), (c-**b**)–E–(o-**a**) and (c-**b**)–E–(o-**c**) could be retained as potential candidates for full-closure using the orbital analysis. The first is quite “exotic” (w.r.t. synthesis of DAEs), but the latter two are built with well-known monomers and they successfully passed the FC orbital topology test in the coupled limit and the curve-crossing test in the weak coupling limit.

## 5 Conclusions

Obtaining efficient multi-photochromic multimers remains an important challenge, especially for diarylethenes. Indeed, while it is, in most cases, easy to induce the first photochromic reaction leading to the closing of one DAE, the remaining open DAEs in the multimers are generally photo-inactive. Aiming to rationalize this long-standing problem, we have used a curve-crossing model considering two DAE switches in the weak-coupling limit. Despite its apparent simplicity, this model clearly demonstrates the emergence of *extra* crossing points, specific to the second cyclization step. These points, formally corresponding to an energy transfer from the reactive closed–open* structure to the unreactive closed*–open form, should be crossed to reach the targeted fully closed isomer. We have provided an analytical formula allowing to calculate the tilt of this crossing on the basis of easily accessible data determinable for the individual DAE, namely the relative stabilities of the two isomers as well as their vertical transition energies. Computing this tilt criterion, we screened a large number of homo- and hetero-dyads. It turned out that the substitution of the reactive carbon atoms, that is known to yield a FC state with a valuable topology to control switching,^33^ delivers quite poor tilts, stressing the challenge of DAE multimers. By contrast, using furan rings and/or a maleimide bridge significantly improved the tilt. Eventually, in an effort to demonstrate the complementarity between this new tilt criterion and previous studies relying on characterization of the Franck–Condon topology, we proposed a series of dyads (Fig. 6) that are most probably worth considering for future synthetic efforts in the field, as they successfully fulfill the two criteria.

Due to the intrinsic complexity of the investigated problem, the present theoretical effort is certainly not the terminus. Our short-term plans include (i) designing a more sophisticated model accounting for the explicit effect of the bridge in the form of an electronic coupling that could alter conjugation between the fragments; (ii) assessing the ET nature and efficiency using alternative models.^54^–^56^ Later, we also plan to account for the possible electronic excitations partly-localised on the bridge.

## Supplementary Material

Supplementary information
