# Supplementary material for: A curve-crossing model to rationalize and optimize diarylethene dyads
Source: Chem Sci. 2015 Jun 29;6(10):5695–702. doi: 10.1039/c5sc01960e (PMC5975843; doi:10.1039/c5sc01960e)
Supplement: Supplementary file 1 [file SC-006-C5SC01960E-s001.pdf]

Electronic Supplementary Material (ESI) for Chemical Science.
This journal is © The Royal Society of Chemistry 2015

Journal Name

ARTICLE TYPE

Cite this: DOI: 10.1039/xxxxxxxxxx

A curve-crossing model to rationalize and optimize diarylethene dyads: ESI

Benjamin Lasorne, Arnaud Fihey, David Mendive-Tapia and Denis Jacquemin

S1 Analytical forms for the energies of the states

Table S-1 Energies of the four ground-state minima for states CC, CO, OC, and OO expressed in terms of the energy parameters of single DAE units.

	H^0_{CC}	H^0_{CO}	H^0_{OC}	H^0_{OO}
oo	$T_A^{\mathrm{O}} + T_B^{\mathrm{O}}$	T_A^{O}	T_B^{O}	0
co	$D_A^{\mathrm{CO}} + T_B^{\mathrm{O}}$	D_A^{CO}	$D_A^{\mathrm{CO}} + T_A^{\mathrm{C}} + T_B^{\mathrm{O}}$	$D_A^{\mathrm{CO}} + T_A^{\mathrm{C}}$
oc	$T_A^{\mathrm{O}} + D_B^{\mathrm{CO}}$	$T_A^{\mathrm{O}} + D_B^{\mathrm{CO}} + T_B^{\mathrm{C}}$	D_B^{CO}	$T_B^{\mathrm{C}} + D_B^{\mathrm{CO}}$
cc	$D_A^{\mathrm{CO}} + D_B^{\mathrm{CO}}$	$D_A^{\mathrm{CO}} + D_B^{\mathrm{CO}} + T_B^{\mathrm{C}}$	$D_A^{\mathrm{CO}} + T_A^{\mathrm{C}} + D_B^{\mathrm{CO}}$	$D_A^{\mathrm{CO}} + T_A^{\mathrm{C}} + D_B^{\mathrm{CO}} + T_B^{\mathrm{C}}$

S2 Results for heteodyads

Table S-2 Tilt criterion (in eV) for the formation of (c-A)–X–(c-B) from (c-A)–X–(o-B)* for various asymmetric DAE A–X–B. dyads

	A																						
B	**a**	**b**	**c**	**d**	**e**	**f**	**g**	**h**	**i**	**j**	**k**	**l**	**m**	**n**	**o**	**p**	**q**	**r**	**s**	**t**	**u**	**v**	**w**
**a**	2.13	2.62	2.37	1.98	1.94	1.96	2.06	1.92	2.12	1.96	1.87	2.23	2.04	2.02	2.05	1.93	2.42	2.18	2.41	2.08	2.28	2.05	2.37
**b**	1.52	2.09	1.80	1.35	1.31	1.33	1.45	1.29	1.51	1.33	1.23	1.63	1.42	1.40	1.43	1.30	1.86	1.58	1.84	1.46	1.69	1.43	1.80
**c**	1.84	2.36	2.10	1.68	1.64	1.67	1.78	1.63	1.83	1.67	1.57	1.95	1.75	1.73	1.76	1.63	2.15	1.90	2.14	1.79	2.00	1.76	2.10
**d**	2.20	2.68	2.44	2.05	2.01	2.03	2.13	1.99	2.19	2.03	1.94	2.29	2.11	2.09	2.12	2.00	2.49	2.25	2.47	2.15	2.34	2.12	2.44
**e**	2.07	2.55	2.30	1.91	1.87	1.90	2.00	1.86	2.05	1.90	1.80	2.16	1.97	1.95	1.98	1.87	2.36	2.11	2.34	2.01	2.21	1.99	2.31
**f**	2.22	2.70	2.46	2.07	2.03	2.06	2.16	2.02	2.21	2.05	1.96	2.32	2.13	2.11	2.14	2.02	2.51	2.27	2.50	2.17	2.37	2.14	2.46
**g**	2.35	2.83	2.58	2.20	2.16	2.18	2.28	2.15	2.34	2.18	2.09	2.44	2.26	2.24	2.27	2.15	2.63	2.40	2.62	2.30	2.49	2.27	2.58
**h**	2.23	2.71	2.47	2.08	2.05	2.07	2.17	2.03	2.22	2.07	1.97	2.33	2.14	2.12	2.16	2.04	2.52	2.28	2.51	2.18	2.38	2.16	2.47
**i**	2.25	2.74	2.49	2.10	2.06	2.08	2.19	2.05	2.24	2.08	1.99	2.35	2.16	2.14	2.17	2.05	2.54	2.30	2.53	2.20	2.40	2.17	2.49
**j**	2.14	2.62	2.38	1.99	1.95	1.97	2.07	1.93	2.13	1.97	1.88	2.24	2.05	2.03	2.06	1.94	2.43	2.19	2.42	2.09	2.28	2.06	2.38
**k**	2.14	2.62	2.38	1.98	1.95	1.97	2.07	1.93	2.12	1.97	1.87	2.23	2.04	2.02	2.06	1.94	2.43	2.19	2.41	2.08	2.28	2.06	2.38
**l**	1.31	1.87	1.58	1.14	1.10	1.12	1.24	1.08	1.30	1.12	1.02	1.42	1.21	1.19	1.22	1.09	1.64	1.37	1.63	1.25	1.48	1.22	1.59
**m**	2.02	2.52	2.26	1.86	1.82	1.85	1.95	1.81	2.00	1.84	1.75	2.12	1.92	1.90	1.93	1.81	2.31	2.07	2.30	1.96	2.17	1.93	2.26
**n**	1.12	1.68	1.39	0.94	0.90	0.93	1.04	0.88	1.10	0.93	0.82	1.23	1.01	0.99	1.02	0.89	1.45	1.17	1.43	1.06	1.28	1.03	1.39
**o**	1.10	1.67	1.38	0.93	0.89	0.91	1.03	0.87	1.09	0.91	0.81	1.22	1.00	0.98	1.01	0.88	1.44	1.16	1.43	1.04	1.27	1.01	1.38
**p**	1.09	1.68	1.38	0.92	0.87	0.90	1.02	0.86	1.08	0.90	0.79	1.21	0.99	0.96	1.00	0.86	1.44	1.15	1.43	1.03	1.27	1.00	1.38
**q**	2.16	2.65	2.40	2.00	1.96	1.99	2.09	1.95	2.14	1.99	1.89	2.26	2.06	2.04	2.08	1.96	2.46	2.21	2.44	2.10	2.31	2.08	2.40
**r**	2.33	2.84	2.58	2.17	2.13	2.16	2.26	2.12	2.32	2.15	2.06	2.43	2.23	2.21	2.25	2.12	2.63	2.38	2.62	2.28	2.48	2.25	2.58
**s**	2.15	2.65	2.39	1.99	1.95	1.98	2.08	1.94	2.13	1.97	1.88	2.25	2.05	2.03	2.06	1.94	2.45	2.20	2.43	2.09	2.30	2.07	2.40
**t**	1.52	2.03	1.77	1.35	1.31	1.34	1.45	1.30	1.50	1.34	1.24	1.62	1.42	1.40	1.43	1.31	1.83	1.57	1.81	1.46	1.67	1.43	1.77
**u**	2.47	2.95	2.70	2.32	2.28	2.31	2.41	2.27	2.46	2.30	2.21	2.56	2.38	2.36	2.39	2.28	2.76	2.52	2.74	2.42	2.61	2.39	2.71
**v**	2.06	2.59	2.32	1.90	1.86	1.88	1.99	1.84	2.05	1.88	1.78	2.17	1.96	1.94	1.98	1.85	2.38	2.12	2.36	2.01	2.22	1.98	2.32
**w**	2.42	2.90	2.66	2.27	2.24	2.26	2.36	2.22	2.41	2.26	2.17	2.52	2.33	2.31	2.35	2.23	2.71	2.47	2.70	2.37	2.57	2.35	2.66

This journal is © The Royal Society of Chemistry [year]

S3 TD-DFT analysis of asymmetric doubly-open dyads

In Table S-3 are presented the optical properties of the three different asymmetric dyads under investigation in their open-open state, that are, in all cases, characterised by an intense $S_0 \rightarrow S_1$ mainly consisting in a HOMO→LUMO transition in the UV region.

Table S-3 Description of the $S_0 \rightarrow S_1$ excited state of selected fully open dyads. In addition to the wavelength (λ in nm) and oscillator strength (f), the localization of the LUMO photochromic orbital is also given, together with the orbital composition of this transition.

Dyad	Bonding interaction	λ	f	Composition
(o-**a**)-E-(o-**b**)	centred on **b**	362	1.00	H→L (89%)
(o-**a**)-E-(o-**c**)	centredon **c**	322	0.97	H→L (57%)
(o-**b**)-E-(o-**c**)	centred on **c**	362	0.99	H→L (89%)

In all cases the LUMO is a photochromic orbital as a bonding interaction between the reactive carbon atoms is found on one of the DTE unit (see Figure S-1). Consequently, it is possible to assess the site of the first ring closure reaction induced by the HOMO→LUMO transition by simply considering the spatial localization of the LUMO photochromic orbital. This LUMO is localized on both the ethynyl linker and on one side of the dyad so that only one unit presents the correct topology at the reactive carbon atoms: that is, **b** in (o-**a**)-E-(o-**b**) and **c** in both (o-**a**)-E-(o-**c**) and (o-**b**)-E-(o-**c**). The products of the first cyclization are therefore expected to be the (c-**b**)-E-(o-**a**), (c-**c**)-E-(o-**a**) and (c-**b**)-E-(o-**c**) mixed isomers.

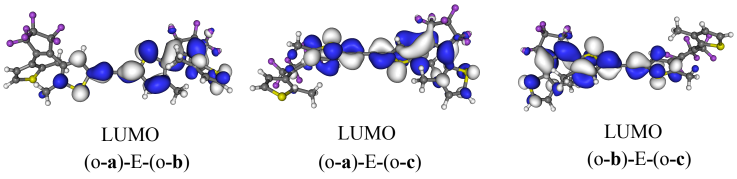

Fig. S-1 Photochromic virtual orbitals for the doubly-open asymmetric dimers.

S4 TD-DFT analysis of selected open-closed isomers

Table S-4 details the optical properties of (c-**qr**)-E-(o-**qr**) (c-**b**)-E-(o-**a**), (c-**c**)-E-(o-**a**) and (c-**b**)-E-(o-**c**).

Table S-4 Photochromic electronic transitions in the open-closed isomers: nature of the photochromic orbital (PO), numbering of the state, absorption wavelength, oscillator strength and ratio of population of the photochromic orbital within this transition. Transitions considered as efficient to induce closure of the second DAE are in bold.

	PO	state	λ	f	population ratio (%)
(c-**qr**)-E-(o-**qr**)	**L**	$\mathbf{S_2}$	**373**	**0.15**	**72**
		S_6	308	0.52	27
(c-**b**)-E-(o-**a**)	**L+1**	$\mathbf{S_2}$	**310**	**0.81**	**17**
		S_4	281	0.22	35
(c-**c**)-E-(o-**a**)	L+1	S_5	285	0.15	37
(c-**b**)-E-(o-**c**)	**L+1**	$\mathbf{S_3}$	**294**	**0.22**	**76**

S5 Influence of the type of conjugated linker in (c-qr)-X-(o-qr)

In Table S-5 we present the optical properties of dimers built with the **qr** unit and two additional types of conjugated linkers, namely a phenyl leading to (c-**qr**)-Ph-(o-**qr**) and a di-ethynyl linker giving (c-**qr**)-E-E-(o-**qr**) (see Figure S-2). The outcomes concerning the photochromic transition are similar to the previous (c-**qr**)-E-(o-**qr**) case. The photochromic orbital is the LUMO for (c-**qr**)-Ph-(o-**qr**) and the LUMO+1 for (c-**qr**)-E-E-(o-**qr**), with very similar topologies (see Figure S-2). The photochromic transition is in both cases the $S_0 \rightarrow S_2$ transition and it presents characteristics that are again similar to those of the ethynyl linker. Those two dimers are expected to undergo the second cyclization as efficiently as in (c-**qr**)-E-(o-**qr**).

Table S-5 Photochromic electronic transitions in (c-**qr**)-Ph-(o-**qr**) and (c-**qr**)-E-E-(o-**qr**). See caption of Table S-4 for more details.

	PO	state	λ	f	population ratio (%)
(c-**qr**)-Ph-(o-**qr**)	**L**	$\mathbf{S_2}$	**378**	**0.18**	**77**
(c-**qr**)-E-E-(o-**qr**)	**L+1**	$\mathbf{S_2}$	**372**	**0.28**	**63**

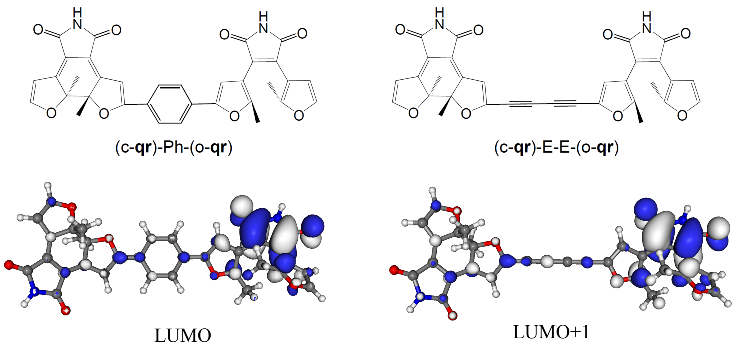

Fig. S-2 Lewis representation and photochromic virtual orbitals for (c-**qr**)-Ph-(o-**qr**) and (c-**b**)-E-E-(o-**a**) dyads.
